# *In Vitro* Evaluation of Proliferation and Migration Behaviour of Human Bone Marrow-Derived Mesenchymal Stem Cells in Presence of Platelet-Rich Plasma

**DOI:** 10.1155/2019/9639820

**Published:** 2019-04-09

**Authors:** Anh Thi Mai Nguyen, Ha Le Bao Tran, Thuy Anh Vu Pham

**Affiliations:** ^1^Faculty of Odonto-Stomatology, University of Medicine and Pharmacy at Ho Chi Minh City, Ho Chi Minh City, Vietnam; ^2^Department of Physiology and Animal Biotechnology, Faculty of Biology-Biotechnology, University of Science, Vietnam National University, Ho Chi Minh City, Vietnam; ^3^Department of Periodontology, Faculty of Odonto-Stomatology, University of Medicine and Pharmacy at Ho Chi Minh City, Ho Chi Minh City, Vietnam

## Abstract

**Objective:**

To access the effects of platelet-rich plasma (PRP) on the behaviour of human bone marrow-derived mesenchymal stem cells (hBMSCs), including proliferation and migration.

**Methods:**

PRP was diluted with DMEM/F12, resulting in concentrations of 1%, 2%, and 5%. The proliferation of hBMSCs was examined by 2 methods: cell-number counting with the haemocytometer method and the colony-forming unit-fibroblast (CFU-F) assay. Cell migration was evaluated using the scratch wound healing (SWH) assay; after that, the recorded digital images were analysed by the Image-Analysis J 1.51j8 software to compare the cell-free areas between groups after 0, 24, and 48 hours.

**Results:**

hBMSCs cultured in DMEM/F12 at PRP concentrations of 1%, 2%, and 5% were all able to proliferate and migrate. In the 5% PRP group, hBMSCs proliferated greatly with a significantly higher cell number than reported for all other groups on days 5, 7, and 9. CFU-Fs were observed in all groups, except for the negative control group. The SWH assay demonstrated that hBMSCs cultured in 2% and 5% PRP almost filled the artificial wound scratch and significantly migrated more than those of all other groups at both 24 h and 48 h.

**Conclusion:**

This study indicated that, due to the significant enhancement of cell proliferation and migration, 5% PRP might be the optimal concentration that should be used to promote the potential of hBMSCs in wound healing.

## 1. Introduction

Periodontitis is one of the most common oral diseases that can be related to the general health and quality of life of patients. With its complicated pathogenesis, it is considered to be a major problem for community health [1]. In the past few decades, the main objective of periodontitis treatment has changed from repairing tissue to regenerating it, thereby reversing the tissue destruction process caused by the disease [2]. Periodontal regeneration is a complex procedure. For that, traditional treatments including scaling and root planing can hardly produce satisfactory results [3]. In these recent years, the using of growth factors has demonstrated outstanding therapy outcomes and has been seen as a promising approach for periodontal tissue regeneration [4].

New materials with the aim of promoting and stimulating the wound-healing process have been continuously studied for application. One of those is platelet-rich plasma (PRP). PRP is defined as a platelet concentration that is 3-4 times higher than normal, collected by the centrifugation of autologous blood once or twice. PRP contains a large amount of growth factors such as platelet-derived growth factors (PDGFs), transforming growth factors (TGFs), and vascular endothelial growth factors (VEGFs). Therefore, PRP has many positive impacts on tissues and cells including angiogenic, mitogenic, and proliferative abilities [4].

On the contrary, with the development of microbiology, stem cells are being researched for application in periodontal regeneration; this includes mesenchymal stem cells (MSCs), embryonic stem cells (ESCs), and induced pluripotent stem cells (iPSCs) [5]. Lee et al. successfully isolated human bone marrow-derived mesenchymal stem cells (hBMSCs) from the mandible [6]. These cells can differentiate into osteoblasts, chondrocytes, myocytes, adipocytes, and vascular smooth muscle cells [7]. Therefore, hBMSCs, with their particular capabilities, seem to be potential materials in periodontal regeneration, especially in alveolar bone formation.

Positive impacts of PRP on the biological behaviours of hBMSCs are undeniable with the proof of many previous studies. Fernandes et al. and Choi et al. concluded that PRP has definite effects on the osteogenic ability of hBMSCs [8, 9]. In those studies, different concentrations of PRP were chosen to determine the optimal PRP concentration for tissue regeneration. The PRP concentration dependence of the biological features of hBMSCs has been proven in many studies such as those by Yamakawa et al. and Amable et al. [10, 11]. Yamakawa et al. stated that the proliferative response of BMSCs in PRP with different concentration is distinctive. However, this dependence is not constant, which means that rising PRP concentrations do not always stimulate cell proliferation and migration [11]. In Amable's experiment, in the 20% and 30% groups, hBMSCs seemed to be repressed and the cell number was significantly lower than that in the control group. In the 40% and 50% PRP groups, cells died immediately, suggesting that high concentrations of PRP might inhibit cell proliferation [10]. Indeed, recently, researchers tended to perform experiments with lower doses of PRP. The experiments of Tavassoli-Hojjati et al. and Jalowiec et al. showed remarkable effects for PRP at low concentrations in comparison with high concentrations [12, 13]. However, in these studies, there was no comparison between low concentrations of PRP, so the optimal concentration of PRP was not determined. Our experiments conducted with concentrations of 1%, 2%, and 5% PRP might be the first to compare the effects between these low concentrations and identify the optimal concentration for the cellular features of hBMSCs, including proliferation and migration. The results of our study might offer accessible information and a constructive foundation for further research into the use of autologous biological materials in periodontal treatment in the future.

## 2. Materials and Methods

### 2.1. hBMSC Culture

hBMSCs at passage 3 were provided by the Physiology and Animal Biotechnology Department, University of Natural Science of Ho Chi Minh City. These cells were then cultured to passage 4 in complete medium (Dulbecco's modified eagle medium: nutrient mixture F-12 (DMEM/F12; Sigma-Aldrich, MO, USA)) supplemented with 10% foetal bovine serum (FBS; Sigma-Aldrich, MO, USA), 100 *μ*g/mL streptomycin (Sigma-Aldrich, MO, USA), and 100 IU/mL penicillin (Sigma-Aldrich, MO, USA) at 37°C and 5% CO_2_. After cells reached 80% confluence, they were ready for the experiments [14].

### 2.2. PRP Preparation

Human peripheral blood was donated by healthy, nonsmoking volunteers aged from 20 to 30 years old. Blood was then centrifugally processed at 2000 rpm for 10 minutes in 3 sterile tubes (8.5 ml/tube). The upper yellow solution of each tube was separated and transferred into another sterile tube and centrifuged the second time at 3500 rpm for 5 minutes. After that, the upper yellow solution (platelet-poor plasma) was removed, resulting in approximately 6 ml of PRP. Calcium chloride was added to PRP for activation. After 15 minutes, pellets were formed and then removed. The tube finally contained pure and activated PRP solution. PRP was diluted with DMEM/F12 to achieve concentrations of 1%, 2%, and 5% PRP for the experimental groups. This research was approved by the Ethical Committee of the University of Medicine and Pharmacy in Ho Chi Minh City with protocol number 225/DHYD-HD [14].

### 2.3. Effects of PRP on hBMSC Proliferation

Two experiments were carried out to evaluate the role of PRP in hBMSC proliferation: (1) the cell-number counting assay with haemocytometer and (2) colony-forming unit-fibroblast (CFU-F) assay. Experimental groups are composed of DMEM/F12 media with 1%, 2%, and 5% PRP. The positive and negative control groups are DMEM/F12 diluted with and without 10% FBS, respectively, for comparison with the experimental groups.

#### 2.3.1. Cell-Counting Assay by Haemocytometer

hBMSCs were cultivated in a 96-well plate (10^4^ cells/well). Culture medium was then replaced by PRP experimental media after 24 hours of culture and continuously cultured for the next 1, 3, 5, 7, and 9 days. At each indicated time point, cells were detached by trypsinization (0.25% trypsin/ethylenediaminetetraacetic acid) and counted using a haemocytometer to determine the number of cells. The cell number was recorded and compared.

#### 2.3.2. CFU-F Assay

hBMSCs were cultivated in a 6-well plate (500 cells/well). Culture medium was replaced by PRP experimental media and cultured on alternating days. At day 16, colonies in the wells were stained with a mixture of 6% glutaraldehyde and 0.5% crystal violet and observed. CFU-F was counted and then compared [14, 15].

### 2.4. Effects of PRP on hBMSC Migration

The scratch wound healing (SWH) assay imitating cell migration during wound healing *in vitro* is relevant for analysing the effect of PRP on cell migration. hBMSCs were seeded into 6-well plates (2 × 10^4^ cells/dish) and cultured until 80% confluence. A scratch was formed in the monolayer on each dish using a sterile pipette tip. Nonadherent cells were removed by washing once with PBS (phosphate-buffered saline). Cells were starved overnight with complete DMEM/F12 and then PRP experimental media was added to the dishes, with each cultured for 48 hours. At time points 0, 24, and 48 hours, cell migration into the empty scratch surface in different experimental groups was observed using a phase-contrast microscope. Images were then recorded by a digital camera and finally analysed using the Image-Analysis J1.51j8 software (Wayne Rasband, National Institute of Mental Health, Bethesda, MD, USA). Cell-free areas in each group recorded at each time point were calculated and compared [14].

### 2.5. Data Analysis

All experiments were repeated at least 3 times. For statistical analysis, independent sample comparison *t*-test and one-way ANOVA with the Dunnett T3 post hoc test (for unequal variances) or the Tukey HSD post hoc test (for equal variances) were used for comparison between groups using SPSS v.22 (IBM, New York City, NY, USA) with a level of significance of 0.05 [14].

## 3. Results

### 3.1. Effects of PRP on hBMSC Morphology

**Figure 1:**
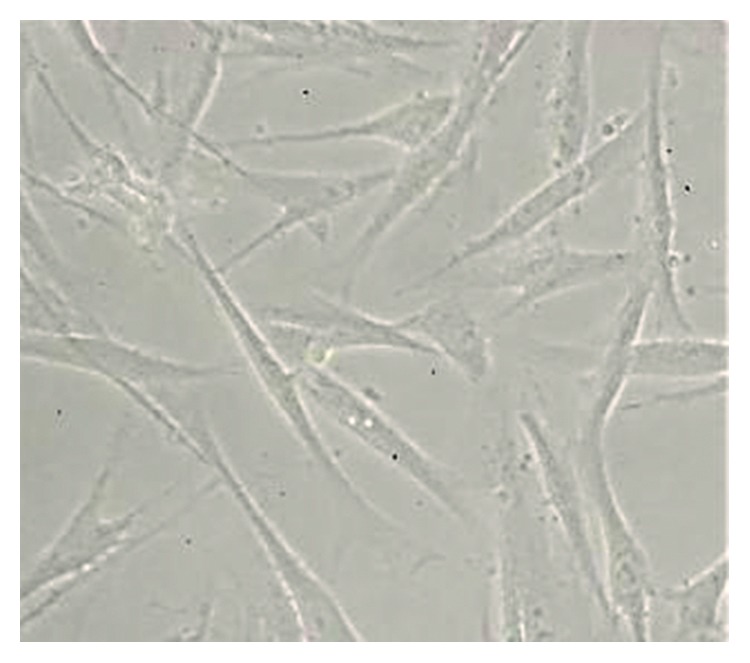
hBMSC morphology cultured in PRP observed under a phase-contrast microscope with ×40 magnification.

hBMSCs were cultured from passage 3 to passage 4 in PRP experimental media at 37°C and 5% CO_2_. Immediately after being cultured, under a phase-contrast microscope, hBMSCs seemed to shrink, develop a round shape, and hover in the culture medium. After 24 hours of culture, cells started to attach to the surface, and after 7–10 days, cells filled most of the cultivating areas with 80–90% confluence. hBMSCs then had an elongated shape with a branched cytoplasm surrounding a round, large nucleus (Figure 1). These morphological features were maintained after many passages. Thus, during cultivating and experimental periods with PRP, hBMSCs showed homogeneity and managed to sustain this through further passages.

### 3.2. Effects of PRP on hBMSC Proliferation

**Figure 2:**
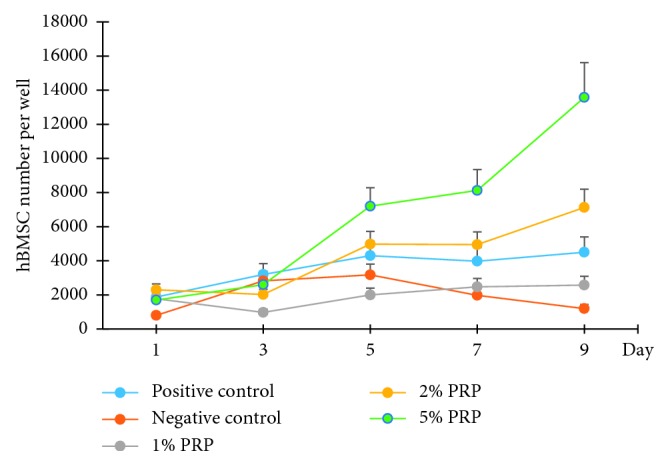
hBMSC number cultivated in experimental groups at days 1, 3, 5, 7, and 9.

In general, hBMSCs were able to proliferate normally in all cultivating media, including PRP experimental groups and control groups. However, the propensity of that in each group differed from each other (Figure 2).

In the negative control group, hBMSCs showed the normal growth of a cell population, consisting of 4 phases: lag (day 1), exponential (day 1 to day 3, cell number increased significantly during this phase (*p* < 0.001)), stationary (day 3 to day 5), and death (day 5 to day 9, cell number decreased significantly during this phase (*p* < 0.01)) (Figure 2).

The growth tendency of the positive control group and 1% PRP group was quite similar. Cell number increased significantly from day 3 to day 5 (*p* < 0.05). However, cell number did not decrease immediately, like the negative control, but stabilised in the following days. There was no difference between cell number at day 5, 7, and 9 in each of these 2 groups (Figure 2).

The proliferation of hBMSCs in the 2% and 5% groups was compelling. Cell number continued to escalate until day 9. In detail, in the 2% PRP group, the number of cells at day 5 was significantly higher than that at day 3 (*p* < 0.001). After day 5, the cell number continues to rise; however, there was no significant difference. In the 5% PRP group, cell quantity significantly escalated from day 3 to day 5 (*p*=0.001) and from day 7 to day 9 (*p* < 0.001) (Figure 2). In brief, hBMSCs cultured with PRP showed a prolonged proliferation period compared to the control group, especially in the 2% and 5% PRP groups.

**Figure 3:**
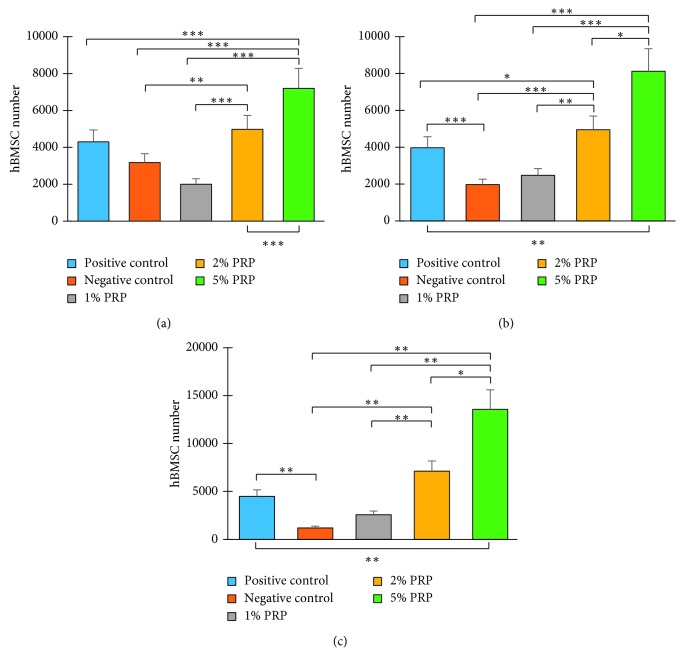
hBMSC number in experimental groups at days (a) 5, (b) 7, and (c) 9 (^*∗*^*p* < 0.05; ^*∗∗*^*p* < 0.001; ^*∗∗∗*^*p* < 0.001).

When comparing the cell number in each group at the same time point, significant differences were also observed, suggesting a different response of hBMSCs in each experimental group. On day 9, cells cultivated with 1% PRP increased in number significantly compared to those in the negative control group (*p*=0.007). At the PRP concentration of 2%, cell number was higher than that in the 1% PRP group on most counting days (*p* < 0.01). In particular, in the 5% PRP group, hBMSCs proliferated greatly with a significantly higher cell number than in all other groups at days 5, 7, and 9 (*p* < 0.05) (Figure 3). This suggested that 5% PRP might be the optimal concentration for this experiment.

**Figure 4:**
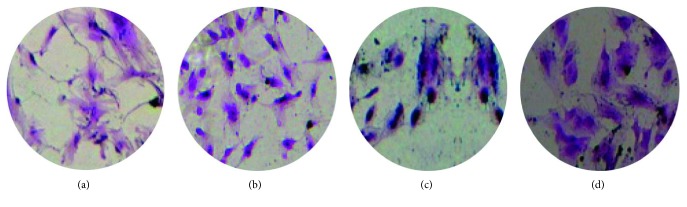
Clonogenic assay at day 16 observed by a phase-contrast microscope with ×10 magnification. (a) Positive control. (b) 1% PRP. (c).2% PRP. (d) 5% PRP.

**Figure 5:**
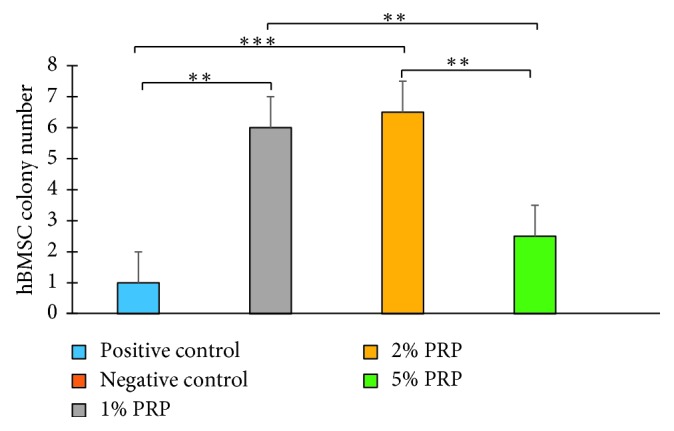
Colony number in experimental groups at day 16 (^*∗∗*^*p* < 0.01; ^*∗∗∗*^*p* < 0.001).

On day 16, except for the negative control group, CFU-Fs were observed in all other groups (Figure 4). In this experiment, the 1% and 2% PRP groups showed better results, with a significantly higher number of CFU-F than in all other groups (*p* < 0.01). Colony number counted in the 5% PRP group was higher than that in the positive control group; however, the difference was not significant (*p*=0.484) (Figure 5).

### 3.3. Effects of PRP on hBMSC Migration

**Figure 6:**
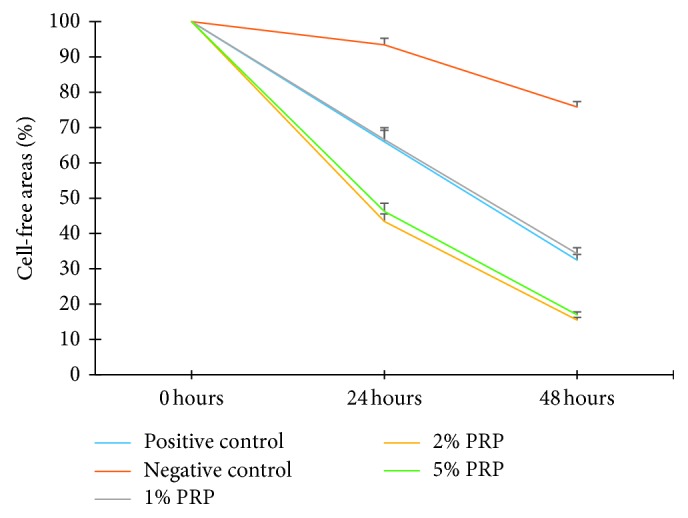
Cell-free area percentage in experimental groups at 24 and 48 h time point. In all groups except for the negative control group, cell-free areas were significantly different at 0 h, 24 h, and 48 h (*p* < 0.001).

**Figure 7:**
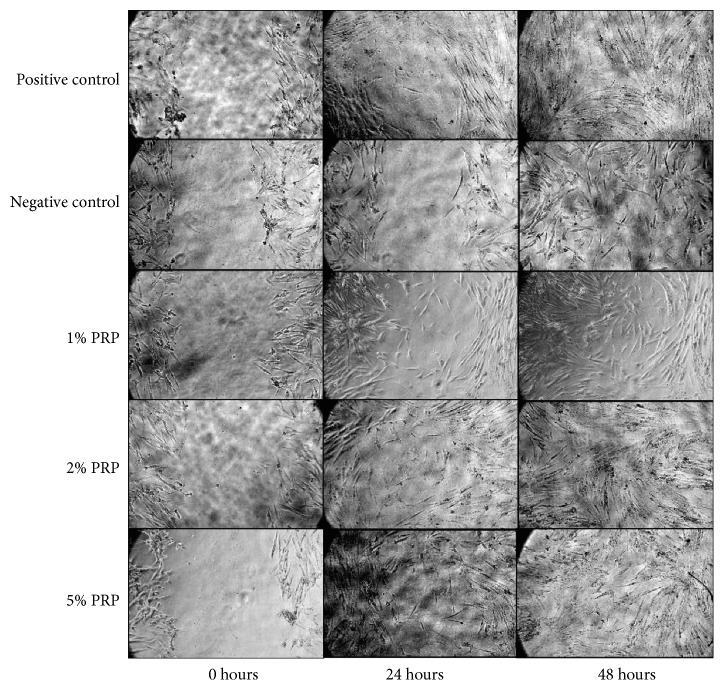
Scratch wound healing assay at 0, 24, and 48 h time points (original magnification of these graphs is ×10).

The effect of different concentrations of PRP (1%, 2%, and 5%) on the migration of hBMSCs was evaluated quantitatively by analysing the reduction in cell-free areas in the SWH assay, mimicking the wound healing process. After 24 hours, except for the negative control group, all experimental groups and the positive control group exhibited cell migration with a significant decrease in cell-free areas (*p* < 0.001). After 48 hours, cell migration was recorded in all groups, including the negative control group, through a significant decline in cell-free areas compared with those at the 24 h time point (*p* < 0.001). Hence, the propensity of cell migration is similar in all groups, which means that cells in all groups tend to fill the wounded areas after being scratched (Figures 6 and 7).

Nonetheless, each group affected cell migration at distinctive levels. At both the 24 h and 48 h time points, 2% PRP and 5% PRP had the narrowest cell-free areas and significantly lower levels than all other groups (*p* < 0.001). There was no significant difference between these 2 groups. There was also no significant difference between the 1% PRP group and the positive control group. These 2 groups had cell-free areas which were significantly narrower than the negative control group (*p* < 0.001) (Figure 6). Accordingly, it could be interpreted that 2% and 5% PRP stimulated hBMSC migration with the best outcomes, even better than standard cell culture medium.

## 4. Discussion

In recent years, PRP usage has become more and more prominent and has been widely applied. PRP is believed to provide growth factors for wounds, thereby accelerating the wound healing process. Many studies have been carried out to reinforce the authenticity of PRP application and achieved conspicuous results [16, 17]. However, these studies used different methods and procedures to obtain PRP. In our research, the PRP used was activated by Ca^2+^. As a result, PRP can regulate almost all growth factors in it. It is quite easy for many cytokines and growth factors in PRP to be restrained and become ineffective. Inactivated PRP takes time to release these molecules, consequently diminishing their effectiveness [18]. The efficiency of the combination of PRP and hBMSCs has been declared in many research studies [19, 20]. According to Lian et al., regarding the combinative efficacy of PRP and BMSCs in diabetic rat wound healing, the group treated with PRP and BMSCs showed significantly better results than the group treated with only PRP or BMSCs and the control group [21].

In this study, changes could be observed in hBMSC number in different groups at different time points. On day 1, the number of cells in the negative control group was significantly lower than that in the other 4 groups. Hence, cells in the PRP experimental groups proliferated more than those in the negative control group, initially proving the ability of PRP to boost cell development. The question is whether cellular response is any different with different concentrations.

On day 3, cell number in the 1% and 2% groups slightly decreased. This could be explained as PRP is a new growth factor in cell culture medium. Therefore, hBMSCs need to become familiarised before proliferating and developing normally [22]. On the contrary, hBMSCs in the 5% PRP group still increased, although not significantly compared to others, but results were still remarkable. This suggests that hBMSCs seem to adapt better to the concentration of 5% PRP than other concentrations.

Day 5 can be considered to be the stationary phase of hBMSCs in the negative control group before declining in quantity at day 7. In the positive control and the 1% PRP group, this stage was prolonged until day 9. The similarity of proliferation in the positive control and 1% PRP groups is related to the results of Lucarelli et al. In their experiment, they compared the proliferation of mesenchymal stem cells in media with 1% and 10% PRP and the control group. The results showed that there was no significant difference between the 1% PRP and control groups [23]. In contrast, in our research, the stationary phase of hBMSCs in the 2% and 5% groups could not yet be monitored. The fact that 5% PRP encouraged the proliferation of hBMSCs better than the positive control group is similar to the results of Goedecke et al. They compared the proliferation of hBMSCs in different media: with 10% FBS, 5% PRP, and 10% PRP. After 8 days, cell counts were performed and the results were as follows: cell number in the 5% and 10% PRP groups increased by 7.4 and 7.7 times, respectively, while cell number in the 10% FBS group was only 3 times higher compared to the beginning [24].

On day 7, cell proliferation in the 2% and 5% groups seemed to slow down. Cell number still increased; however, the rate was not as substantial. A possible explanation is that as the cells grow, they will compete with each other to survive. As a result, weaker cells will perish, leaving more tenacious cells to keep proliferating [22].

Finally, on day 9, cell number in the 5% PRP group accelerated significantly higher than the other groups. Cell quantity in the 2% PRP group was also notably higher than in the negative control group and 1% PRP group. Hence, we can see diverse responses of hBMSC proliferation to different concentrations of PRP. Previous studies had surveyed the PRP concentration dependence of cell proliferation. For instance, Yamakawa et al. prepared different concentrations of PRP to inspect the proliferation of rat BMSCs on days 1, 2, 3, 4, and 5. They finally confirmed that the proliferative response of BMSCs in different concentration of PRP is distinctive [11]. However, this dependence is not consistent, which means that increasing PRP concentrations do not always stimulate cell proliferation. This theory was also demonstrated by Amable et al. [10]. Different concentrations of PRP were used, including 1%, 2.5%, 5%, 10%, 20%, 30%, 40%, and 50%, and BMSC proliferation was checked every 20 hours. The results showed that cells in 10% PRP have the highest proliferation rate, while hBMSCs seemed to be repressed in the 20% and 30% groups, with the cell number being significantly lower than that in the control group. In the 40% and 50% PRP groups, cells died immediately, suggesting that high concentrations of PRP might inhibit cell proliferation [10]. This explains why the number of receptors on cells is limited. Therefore, when all of these receptors bind to specific growth factors, the remaining growth factor in the culture medium will become redundant and contaminate cells. As a consequence, optimal platelet concentration depends on the target cells [25].

Indeed, recently, researchers tended to perform experiments with lower doses of PRP. The experiments of Tavassoli-Hojjati et al. showed the remarkable effects of PRP at low concentrations in comparison with high concentrations. In these experiments, 0.1% and 5% PRP were shown to have better effects than 50% PRP [13]. This result is similar to that of Jalowiec et al. It was concluded that 5% PRP is better for hBMSC proliferation than 10% or 50% PRP [12]. Along with this tendency, our experiments were conducted with PRP at concentrations of 1%, 2%, and 5% to compare the effects of these low concentrations and identify the optimal concentration for hBMSC cellular features.

In the CFU-F assay, 5% PRP showed more prominent results in colony forming than the positive control group; however, there was no significant difference. This finding is comparable to that of Goedecke et al. After 14 days of cultivation, the number of colonies recorded was 25, 20, and 16 in the 10% FBS, 5% PRP, and 10% PRP groups, respectively, with no significant difference [24]. Also, in our study, the 1% and 2% PRP groups indicated a better ability for colony forming with significantly higher colony numbers than other groups. The fact that lower concentrations of PRP in this experiment were more effective might be because of the lower cultured cell density (500 cells per well compared to 10^4^ cells per well in the cell-counting assay). Lower density leads to fewer cell receptors, which results in a lower dose of growth factors being needed for cell proliferation [25]. In conclusion, PRP showed a noteworthy ability in cellular stemness, boosted by stimulating colony formation, so high PRP concentrations were not necessary for this experiment.

Many studies have researched hBMSC migration, and the results are relevant to this study. In the experiment by Liu et al., the SWH assay was performed to determine hBMSC migration ability at the 8 and 12 h time points. Cell-free areas were then analysed by Image-J at each time point. The results showed that cell-free areas decreased significantly after 12 hours compared to the control group [26]. The experiment carried out by Cenni et al. also showed analogous results. After 20 hours of culture at 37°C and 5% CO_2_, it can be assumed that hBMSCs migrated better in the medium with PRP than platelet-poor plasma and the control groups [27]. However, these experiments did not compare the efficacy of different concentrations of PRP on hBMSC migration. In our study, the comparison of the 1%, 2%, and 5% groups was carried out to determine the optimal PRP concentration for hBMSC migration. Overall, the propensity of cell migration is similar in all groups, which means that cells in all groups tend to fill the wounded areas after being scratched. However, cell migration in each group was perceptibly different. Thus, we had a quantitative evaluation by the Image-J software to determine how much hBMSCs migrated. It helped to calculate the cell-free areas in each plate at the 0, 24, and 48 h time points. The narrower the area, the more the cells migrated; therefore, comparisons between groups could be carried out. As a result, the 2% and 5% PRP groups stimulated hBMSC migration the most through the narrowest cell-free areas after 24 and 48 hours. Moreover, 1% PRP had the tantamount effect on cell migration to the positive control group.

If we simply perform a scratch wound, cell proliferation and migration to fill the wound might not be identified. This is the disadvantage of this experiment. Aphidicolin or proliferation inhibitors were not used [28] and the serum concentration was not reduced [29] to regulate cell proliferation, as in some previous studies. However, using proliferation inhibitors or reducing serum concentrations is distinctive for each cell, and if there is any inaccuracy in dose, cell behaviours might be uncontrollable, which leads to cell poisoning and death. On the contrary, the cell cycle of hBMSCs in previous studies was recorded to be 34.2 hours [30] or 29.7 hours [31]. Therefore, if cells were starved before the experiments, cells enter the G0 phase of the cell cycle [32]. Here, hBMSCs must take that amount of time to continue duplicating. For that reason, it can be considered that the cell number was stable at the 24 h time point and that the cell-free area being filled is a result of cell migration. Consequently, in this experiment, hBMSC migration results at the 24 h time point are dependable, meaning that 2% and 5% PRP promoted hBMSC migration better than 1% PRP and the control groups.

## 5. Conclusion

Cultured in media with PRP, hBMSCs, without any change in morphology, were shown to be able to proliferate and migrate. However, these behaviours depend on the concentration of PRP. In the cell-counting assay, PRP notably promoted cell proliferation, especially in the 5% PRP group, where cell number outgrew all other groups significantly at days 5, 7, and 9. hBMSCs cultured in 5% PRP also showed remarkable CFU-F forming ability after 16 days. Finally, in the SWH assay, at the 24 h time point, 5% PRP showed the best migration stimulation through the narrowest cell-free areas compared to all other groups. In conclusion, through the significant enhancement of cell proliferation and migration, 5% PRP might be the optimal concentration that should be used to promote the potential of hBMSCs in wound healing.

## Data Availability

The data used to support the findings of this study are available from the corresponding author upon request.
